# On the Use of Opium in Congestion

**Published:** 1878-02-20

**Authors:** A. B. Loving

**Affiliations:** Duval’a Bluff, Arkansas


### ON THE USE OF OPIUM IN CONGESTION.

[Prepared for the Sebastian County Medical Association.]

BY A. B. LOVING, M.D.

As I have been called upon by the Society for a communication on the use of Opium in Congestion, I will endeavor to give my experience with this agent in the treatment of that form of congestion commonly known as congestive chill. If you expect me to give you the *modus operandi* of opium in this disease, I fear you will be disappointed. One thing I do know, I have gained my object in the treatment of this trouble, with this agent in combination with quinine, calomel and camphor, when I do not think I could have reached the same results without opium.

And since comparing my statistics with those of others who do not use opium in congestion, I feel confident that my success in the treatment of this disease, is due largely, to the opium used. I know that some physicians take the ground that opium will not be absorbed while the system is in this state of congestion, and will accumulate in the stomach, and, if the patient is so fortunate as to struggle through the congestive stage, he will be poisoned with opium. I admit, if the opium was not taken up by the absorbents and disposed of by the system, that this state of things might result. But the success of my treatment indicates that absorption does take place.

If opium cannot be absorbed in these cases, I affirm that no other medicinal agent can be. And, admitting this, what is left us to do? Shall we stand by and watch the feeble flame of life go out, or shall we endeavor to rekindle it with opium, the agent which experience has taught us, will snatch our patients, as it were, from death's cold embrace, and restore them to family and friends?

I hold that medicines *can* be absorbed so long as the heart continues to pulsate with sufficient force to propel the life-giving fluid into the primæ viæ, and being absorbed, will produce its peculiar effect on the system; the dose, of course, to be regulated by the state or condition in which we find the patient. Now, we all know what authors say as to the effect of opium. On page 701 *Stillé's Therapeutics*, will be found the following:

"In small doses, from one-fourth to one grain, opium produces on those who are accustomed to its use, a soothing, luxuriant calm of mind and body; followed, in the course of 40 or 50 minutes, with a disposition to sleep, etc., etc. At the same time, the pulse, which was at first slightly quickened, became somewhat slower, and often perspiration breaks out on the skin. In larger doses, as from one to three grains, opium produces much more decided effects; the stage of excitement is much more strongly marked; the head feels full, hot and sometimes light; there are buzzing noises in the ears; the face and eyes are injected; the pulse is fuller and more frequent; the skin is hot and dry. As a general rule a full dose of opium renders the action of the heart stronger and more frequent."

We see from this that the first effect of the opium is to stimulate, and its after effect to depress. During its stimulant action, Stille says: "The skin becomes turgid and red."

I claim for opium that in this congested stage we get the stimulant action without the subsequent depression. I know not how to account for this, unless the quinine and camphor I always give in combination with the opium, maintains the stimulant effect of that most potent drug. At least, experience has taught me that no depression follows in these cases. I will report a few cases as correctly as I can draw on my memory for the same:

*Case* 1.—I was sent for to see Mrs. C., on the morning of ——; found her suffering from pernicious intermitent fever, with congestion of the lungs; respiration hurried, perhaps 45 or 50 to the minute, pulse quick and thready; surface decidedly cool and moist; muscles in a state of perfect relaxation. I felt that I had here a case that would speedily terminate in death, if prompt measures were not taken to avert it. I directed the chest to be enveloped with mustard plasters, and I gave the following medicines: quinine, opium, camphor, acetate of lead, and a small quantity of calomel. I was not particular about measureing the quinine or camphor, and don't think I gave less than one grain, or a grain and a half of opium, perhaps more; when I got it all together I had a large size country teaspoonful. I gave this dose myself, and left 5 or 6 powders of the same preparation, but compounded with more care, and directed one to be given every two hours until the respirations became free and easy, and then to prolong the intervals to five or six hours. On my return the next morning, I found all the powders had been given except, I think, about 2, congestion relieved, patient in every way improved; directed quinine to be kept up at intervals of 4 to 6 hours for the next 48; also directed, if the bowels did not act in the course of the day, to be moved with castor oil. At my next visit, found patient so much improved that I turned her over to the cook.

*Case* 2—was Mr. F., a case of congestion of the bowels, with copious watery discharges which were taking place every 15 or 20 minutes; complete relaxation of the system; pulse quick and compressable. I can't say how much morphine I gave him at the first dose, but poured it out in a spoon, like I was measuring quinine, dissolved it in as little water as it would dissolve in, and gave it forthwith. I then measured out 5 other morphine powders containing about ¼ of a grain, directed them to be given every hour and a half until vomiting and purging were arrested. Also left 20 grains quinine, divided into 5 powders, one to be given as soon as the stomach would retain it, and repeated every 2 hours until all were given. I do not recollect that I gave any calomel in this case, but expect I did, as I hardly ever omit it in these malarial attacks. I do not hesitate to give the mercury in cases when the actions from the bowels are frequent, believing, as I do, that its remote action helps to guard the patient against a repetition of the paroxysm, or congestive stage, and besides, by combining with opium and some astringent medicine, the bowels will be checked and the calomel will be held in the system until it has the desired effect.

*Case* 3—was Mrs. G—, with remittent fever with paroxysmal headache, the pain in the head coming on every 10 or 15 minutes, somewhat after the order of a neuralgic attack; pulse full and bounding; skin hot and dry, tongue heavily coated with a brown coating; bowels costive. This was a case of ordinary remittent fever, except the peculiar form of headache. I directed opium half grain, quinine three grains, calomel two grains, to be given every two hours until relieved, and then every 3 or 4 hours until 5 or 6 doses had been taken. Next day, found her comfortable, head symptoms relieved; directed bowels to be moved with oil, and quinine to be kept up for several days. I report this case not because of the opium used, but the administration of quinine when the pulse was full and hard, and skin hot and dry, with the head trouble that was present. Which we all know would have been contrary to the teachings of the medical profession 10 or 15 years ago.

*Case* 4.—I was called to see Mr. M—, and found a case of pernicious intermittent fever; pulse weak and compressable; skin cold and bedewed with sweat; bowels discharging blood every half hour or hour. I informed the patient's friends that I did not think he would live till morning. I had a large mustard plaster put on the abdomen, gave him 15 grains quinine, 1 grain opium, 5 grains camphor and 3 grains of lead; directed powders to be given every 2 hours until the disease was controlled. This case recovered in two or three days.

Duval's Bluff, Arkansas.

